# A novel approach for audible acoustic quick response codes

**DOI:** 10.1038/s41598-022-09858-7

**Published:** 2022-04-19

**Authors:** Weijun Zhu, Ziang Gao, Yiran Wang

**Affiliations:** 1 School of Computer and Artificial Intelligence, Zhengzhou University, Zhengzhou, China; 2 School of Electronics Engineering and Computer Science, Peking University, Beijing, China; 3 School of Network Engineering, Zhoukou Normal University, Zhoukou, China

**Keywords:** Computer science, Software

## Abstract

Compared to image-based quick response (QR) codes, acoustic QR codes have some advantages. However, an acoustic QR scanner cannot recognize an acoustic QR code at a distance of more than two meters from an acoustic QR announcer. To this end, we propose a new sort of acoustic QR code, called an audible acoustic QR code (AAQRC), which employs humanly audible sound to carry users’ information directly. First, a user’s string of characters is translated into a string of pitches. Then, the related algorithms convert the string of pitches into a playable audio file. As a result, an AAQRC is generated, consisting of the audio itself. AAQRC recognition is the opposite process of AAQRC generation. Compared with the existing approach for acoustic QR codes, the new method can recognize acoustic QR codes at a longer distance, even if there are obstacles between the AAQRC announcer and AAQRC scanner.

## Introduction

QR codes have been used widely and deeply affect people's lifestyles. Generally, the QR process contains two stages: generation and recognition. The principle can be described as follows:

1. During generation, a URL is encoded into a binary string, and each binary character is expressed by a dot in a QR image. For example, a black dot may express binary “1”, and a white dot express binary “0”. Furthermore, the positional relationship among different dots in a QR image is used to express the sequence relationship among different binary characters. For example, the first binary character in the string can be expressed by the dot in the first row and the first column, while the second binary character in this string can be expressed by the dot in the first row and the second column. Thus, a QR image carrying information is formed.
2. During recognition, a user scans a QR image to identify black dots, white dots and their positional relationship. The URL information contained in this QR code is obtained, employing a process opposite to the generation stage.

However, the image-based QR technique has some disadvantages: (1) to avoid a poor effect of scanning, a user has to adjust the angle between his or her camera and a QR image and make them face to face; (2) many external factors may limit the result of scanning, such as brightness; and (3) no obstacle is permitted between the camera and the QR image when a user scans the QR code. To this end, Dagan et al. pioneered an acoustic QR technique called acoustic QR codes, which uses acoustic signals to carry QR information^1^.

An acoustic QR^1^ uses sound waves that cannot be heard by human ears to carry users’ information. First, sound signals expressing users’ information are modulated into a modulated complex lapped transform (MCLT). Then, the MCLT with the sound signal is transmitted outside by an acoustic QR transmitter. A acoustic QR receiver receives the modulated MCLT and uses the demodulation algorithm to separate the sound signal from the MCLT prior to translating it into the user’s information, finishing the process. In this way, the above problems are relieved because sound rather than images are employed to carry users’ information. Of course, it is generally accepted that “QR” means “quick response”, whether by acoustic or by image. You can also call an acoustic QR another name which has nothing to do with “QR” if you like.

The acoustic QR is promising and emerging, but still has some shortcomings: (1) a receiver must be close to the transmitter^1^; (2) the acoustic wave cannot be heard by human ears^1^, so a user is unaware of the existence of the QR codes and his or her unexpected scanning actions.

Let us imagine some potential scenarios. You are shopping in a mall, and you take out your mobile phone and plan to "scan” an acoustic QR code to pay for your purchased goods. Considering that “showing” and “scanning” an acoustic QR cannot be heard and perceived by human ears, how do you know when the acoustic QR begins, when it ends, and whether it is synchronizing and communicating with your mobile phone? Or, you are not shopping, but just happen to walk past someone else. How can you realize whether an acoustic QR is playing a role for his or her payment, which may try to direct your mobile phone to an undesired payment webpage? In addition, what about muting advertising bombardment? What about the silent direction to malicious websites built by hackers? Perhaps you do not realize your mobile phone is trying to access some undesired webpages covertly, due to the voiceless “showing” and “scanning” of an acoustic QR. You may not be aware of the existence of an acoustic QR at all, although it is doing something with your mobile phone.

We therefore have to think about something important. In terms of the image-based QR technique, a user can “see and perceive” when a QR code is being shown and/or scanned. However, in terms of the existing acoustic-based QR technique, a user cannot “hear and perceive” similar actions are taking place, so far. Thus, an audible acoustic QR technique is needed. Motivated by this, we propose a different acoustic QR called an AAQRC.

On the one hand, a URL address is translated into a piano piece, which is the obtained AAQRC. On the other hand, playing this piano piece means that the AAQRC is being shown as a QR code, and listening to the piano piece means that the AAQRC is being scanned as a QR code. As a result, a novel sort of QR codes that directly use humanly audible sound itself as QR codes is pioneered, directly and obviously removing the second shortcoming of the existing acoustic QR mentioned above. Furthermore, our experiments demonstrate that a receiver does not need to be close to its transmitter using an AAQRC, overcoming the first shortcoming of the acoustic QR mentioned above. The combination of the above points forms the contribution of this study.

The remainder of this paper is organized as follows. The “Background” section provides some elementary knowledge. The “The Principle of Audible Acoustic QR Codes” section proposes the new method, including the two algorithms, and analyzes the complexity of these algorithms. The “A Case Study” section discusses a case study. The experiments that were carried out are discussed in the “Experiments” section. The “Comparisons between this work and related ones” section compares related research with this study. The last section draws the conclusions of this paper.

## Background

### MIDI file^14^

The musical instrument digital interface (MIDI) was proposed to address the communication problem between electronic-acoustic instruments. As the most widely used musical standard format, a MIDI is regarded as "a music score understood by a computer". To date, the MIDI has become one of the standard languages used by electronic musical instruments and computers, and an agreement about the set of messages (i.e., instructions). A MIDI itself generates no sound signal. However, it records each musical note as a number and transmits various messages about these numbers in a cable. The electronic-acoustic equipment receiving the message generates sound or performs some actions, according to the message.

Basically, a MIDI file consists of two parts: a block about the file’s header and a block about the audio tracks. The former block includes (1) a subblock identifying the type of file (4 bytes); (2) a subblock indicating the length of the next subblock called the data area of the current block (4 bytes); and (3) a subblock called the data area of the current block (6 bytes).

At the beginning of each MIDI file, the file’s header block has the following hexadecimal string of numbers: "4d 54 68 64 00 00 00 06 ss ss nn nn tt tt". In this string, "4d 54 68 64" is the substring identifying the type of file, and it indicates that this file is a MIDI file. The value of the subsequent substring is “00 00 00 06” because the next subblock, called the data area of the current block, always has six bytes.

The meaning of the first two bytes in substring "ss ss nn nn tt tt" is as follows: "00 00" means that there is only one track; "00 01" means that there are multiple synchronous tracks; "00 10" means that there are multiple independent tracks. In addition, the substring “nn nn” specifies the number of tracks, while the substring "tt tt" specifies the time format and the highest bit is a label. If the value of this bit is 0, tick timing is used. Otherwise, the SMPTE format is employed for timing.

For example, supposing the file’s header string is "4D 54 68 64 00 00 00 06 00 01 00 03 01 E0", it means that (1) this is a MIDI file; (2) it has three synchronous tracks; and (3) it uses tick timing, and each quarter note contains 480 ticks, since 480 in decimal is equal to 1E0 in hexadecimal.

There are one or more blocks about the audio tracks, posterior to the block about the file’s header, in a MIDI file. Each audio track block includes three parts: (1) a subblock identifying the type of track (4 bytes) and track block data area length (4 bytes); (2) a subblock indicating the length of the next subblock called the data area of the current block (4 bytes); and (3) a subblock called the data area of the current block (consisting of multiple MIDI events).

The first subblock is "4d 54 72 6b" in hexadecimal. A MIDI event contains dynamic bytes and MIDI messages. MIDI messages may be channel messages or system messages. Channel messages play a key role in recording music scores. Its main functions include releasing musical notes, pressing musical notes, touching musical notes, changing a controller, changing an instrument, changing a pitch wheel, setting the sequence in a track, event on texts, notice on copyright, designating the name of a song/track, designating the musical instrument, lyrics and notes, termination of track, specifying speed, specifying beat, and so on. For example, let a piano be used; a pitch called C4 will be recorded if one presses C4 at one time and releases this button at the next time.

In this way, a MIDI file records a music score understood by a computer. Ref. **14** provides more details on the MIDI format, helping us understand the principle of translating a string of pitches into a MIDI file and the reverse procedure.

### Measuring pitches using an algorithm

In short, the key principle of this sort of algorithm are as follows.

First, an acoustic sensor is employed to feel the vibrations caused by a pitch. On this basis, the acoustic sensor can measure how much time (T) a vibration requires. Second, let f = 1/T, and f is the frequency of the vibrations. Third, a fundamental frequency determines a pitch, and harmonics determine timbres^15^. Thus, one can determine the value of a pitch with the value of f provided, since there is a rough map relationship between the pitches and frequencies^16^. Fourth, one can obtain the values of all pitches in a piece of MIDI audio by repeatedly executing all three steps mentioned above for each pitch.

## The principle of audible acoustic QR codes


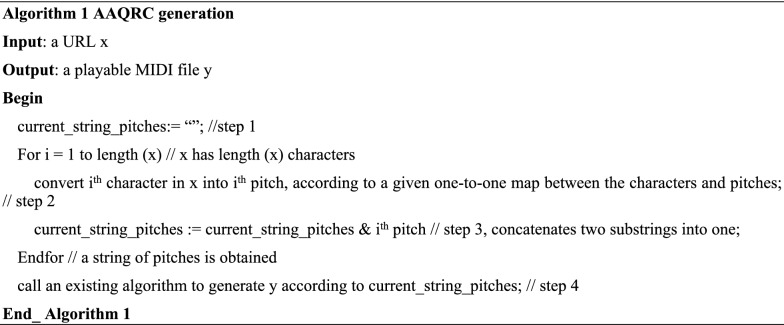


### The principle and the algorithms

In brief, we employ humanly audible audio to directly encode user information in a QR code. The principle of the new approach is as follows.

**Figure 1:**
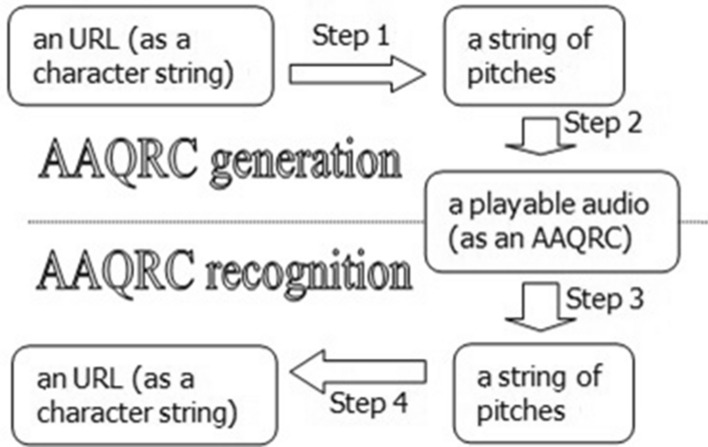
AAQRC generation and its recognition.

First, a one-to-one map between a set of frequently used characters and a set of frequently used pitches is constructed. Thus, a string of characters is translated to a string of pitches, and the latter string is employed to express a URL. As a result, an AAQRC will be generated if a piece of music (such as a piano piece) is generated, whereas this AAQRC will be recognized if this piece of music is played. The new method has four steps, as shown in Fig. 1 and algorithms 1 and 2.

It should be noted that AAQRC recognition has two optional modes/ways: recognizing a file (Mode 1) and playing and listening (Mode 2). The difference is that the MIDI file itself will be recognized with the former mode, while the sound being heard in the air will be recognized with the latter mode.

With Mode 1, step 6 calls an algorithm to translate a MIDI file into a string of pitches, using the procedure mentioned at the end of the “MIDI file” subsection. With Mode 2, step 6 calls an algorithm to translate a series of acoustic signals into a string of pitches, using the procedure mentioned in the “Measuring pitches using an algorithm” subsection.

### Time complexity

Let length(x) = n. Step 1 completes its computational task within O(1) time, as does step 3. If there are m rows in the one-to-one map between the characters and the pitches (m different characters and m different pitches are used), seeking a given character or pitch will take O(m) time, so step 2 will consume O(m) time. In addition, step 4 will take O(n) time, according to the principle of the MIDI file mentioned in the previous section.
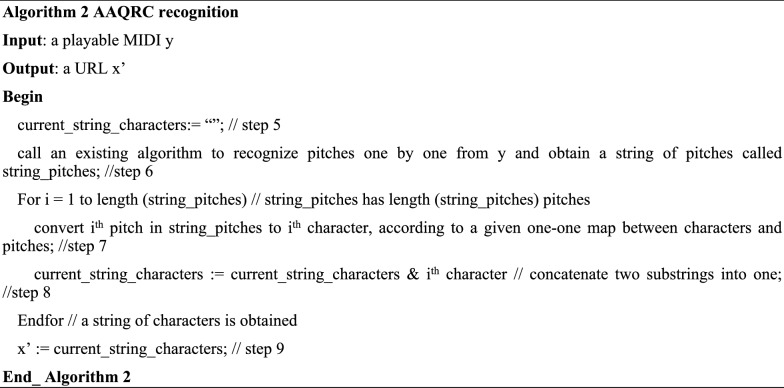


Considering that steps 2 and 3 are executed O(n) times, we can safely say that algorithm 1 consumes O(1) + O(n)*(O(m) + O(1)) + O(n) = O(m*n). This is the complexity of algorithm 1.

Let length(string_pitches) = n. Step 5 can complete its computational task within O(1) time, as can steps 8 and 9. If there are m rows the one-to-one map between the characters and the pitches (m different characters and m different pitches are used), seeking a given character or pitch will take O(m) time, so step 7 will consume O(m) time. In addition, step 6 will take O(n) time, according to the principles of procedures mentioned in the previous section, regardless of whether it is Mode 1 or Mode 2.

Considering that steps 7 and 8 are executed O(n) times, algorithm 2 consumes O(1) + O(n) + O(n)*(O(m) + O(1)) + O(1) = O(m*n). This is the complexity of algorithm 2.

In other words, the proposed algorithms have polynomial complexities, and they can complete their computational tasks in polynomial time.

## A case study

Let us take the official website of Zhengzhou University ("www.zzu.edu.cn") as an example to test the process of AAQRC generation and recognition. Table 1 shows the platform and tools used in our experiments.

**Table 1:** The platform and tools used.

Platform and tool	Function
Pickup: Huishengyue USB-10	Pick up sound
SAST loudspeaker: A90, 15 inches	Play an audio
Overture 5^2^	Execute step 2 of the new method
MidiEditor^3^	Execute step 3 of the new method
Bideyuanli^4^ online tool	Execute step 3 of the new method
Experimental platform establish by us, (see Fig. 2)	Execute step 1 and step 4 of the new method
Sound Meter^5^ app	Test sound volumes at the pickup

**Figure 2:**
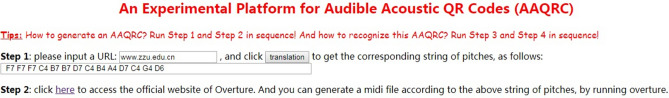
An example on AAQRC generation: step 1.

First, a string of characters, i.e., f1 =  “www.zzu.edu.cn”, is inputted, as shown in Fig. 2, And step 1 in the new method translates f1 into the corresponding string of pitches, i.e., f2 = ”F7 F7 F7 C4 B7 B7 D7 C4 B4 A4 D7 C4 G4 D6”.

**Figure 3:**
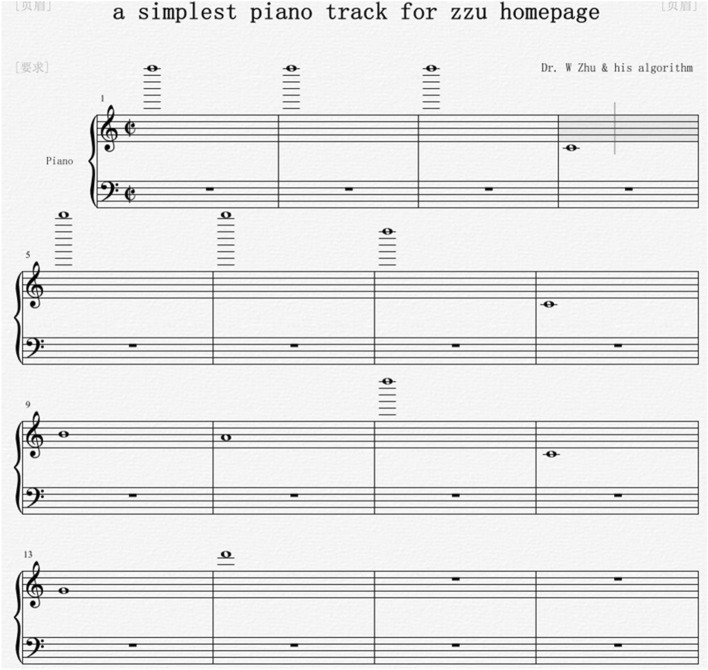
An example on AAQRC generation: step 2: music score of f2.

Then, Fig. 3 illustrates a music score of f2 with Overture 5^2^. Using Overture, we generate a playable MIDI file called “testzzu_h.mid” according to the music score of f2. This MIDI file itself is the produced AAQRC for the homepage of Zhengzhou University.

The process of AAQRC recognition from this audio is as follows.

**Figure 4:**
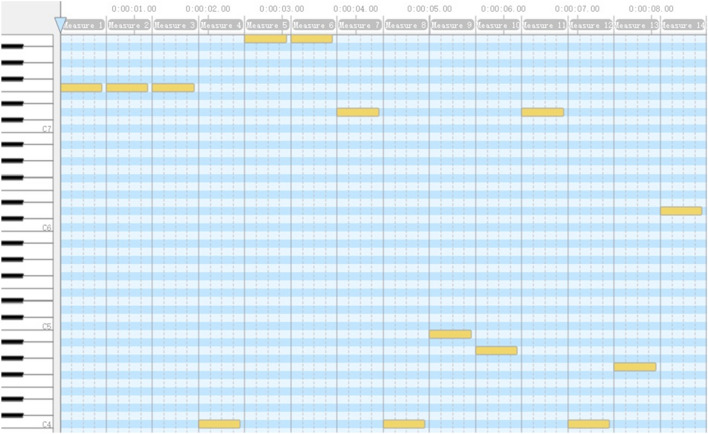
An example on AAQRC recognition: step 3: music score of f3.

First, the string of pitches f3 is read directly from “testzzu_h.mid” using MidiEditor^3^ (Mode 1 is used). As shown in Fig. 4, f3 = "F7 F7 F7 C4 B7 B7 D7 C4 B4 A4 D7 C4 G4 D6". Clearly, f2 = f3 holds.

**Figure 5:**
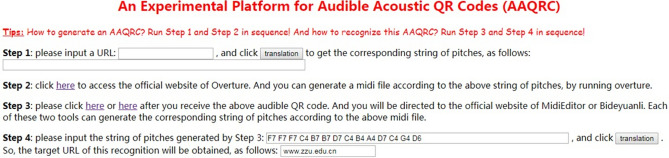
An example on AAQRC recognition: step 4.

Finally, as shown in Fig. 5, the value of f3 is inputted, and step 4 in the new method translates f3 into the corresponding string of characters, i.e., f4 = “www.zzu.edu.cn”. Clearly, f1 = f4 holds, indicating that the recognized URL equals the intended URL. It is clear that AAQRC generation and recognition are successful, in this example.

**Figure 6:**
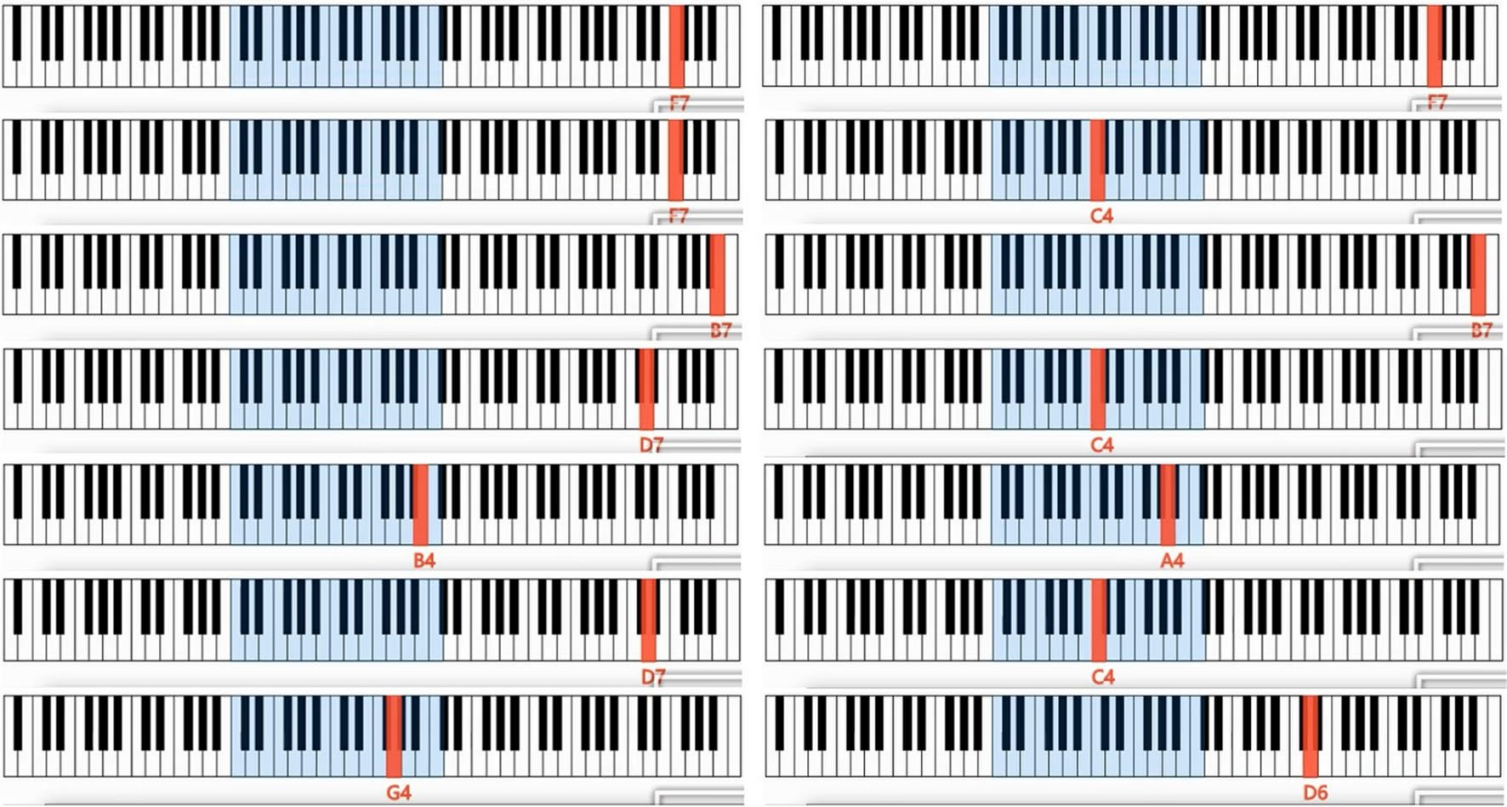
An example on AAQRC recognition: step 3: music score of f3’.

It should be noted that a QR announcer can also show its AAQRC by playing “testzzu_h.mid”, whereas a QR scanner can recognize this AAQRC by listening to this audio (Mode 2 is used). We employ the loudspeaker listed in Table 1 to play the audio at a normal volume and use the pickup listed in Table 1 to pick up the sound. The distance between the pickup and the loudspeaker is set to 3 m, and these two devices are separated by a baffle. An online tool called Bideyuanli^4^ is employed to convert the sound collected by the pickup into a string of pitches f3'. As shown in Fig. 6, f3' = " F7 F7 F7 C4 B7 B7 D7 C4 B4 A4 D7 C4 G4 D6". Clearly, f3 = f3' holds, indicating that all pitches are correctly identified.

## Experiments

### Experimental objective

We aim to explore whether the new method is effective. To be specific, can an AAQRC scanner effectively recognize the URL information sent by an AAQRC announcer at a distance?

### Experimental platform

Please see Table 1. This table depicts the experimental platform used in this study. It should be noted that all the acoustical equipment was selected randomly, without any special consideration.

### Experimental procedure

Step (1). Thirty different URLs are selected randomly, where each of the ten URLs contains ten characters, and each of another ten URLs contains twenty characters, and each of the other ten URLs contains thirty characters.

Step (2). For each of the thirty URLs, we produce the corresponding string of pitches using Overture according to a given relationship between characters and pitches. On this basis, thirty MIDI files are generated.

Step (3). Each of the thirty MIDI files is played on a machine with a loudspeaker, and another machine with a pickup receives the acoustic signals and tries to recognize them at a distance. In other words, Mode 2 is employed since AAQRC recognition in Mode 1 is easier.

Step (4). For each of the thirty MIDI files, the recognized acoustic signals are translated to the corresponding strings of pitches using Bideyuanli.

Step (5). For each of the thirty obtained strings of pitches, the recognized string of characters is obtained according to the given relationship between characters and pitches.

### Experimental results and some discussions

**Figure 7:**
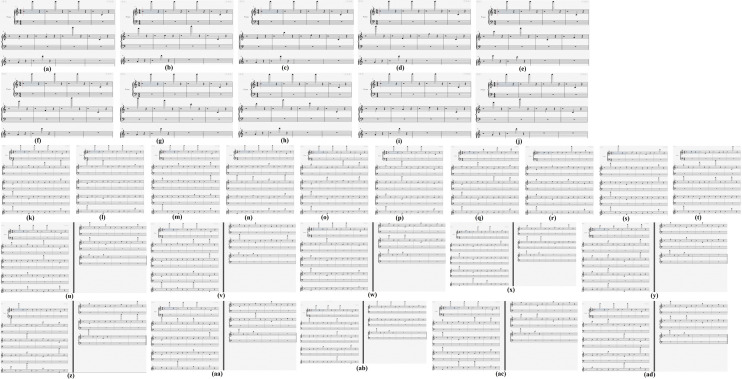
Music scores of the thirty URLs. (**a**) a1.mid; (**b**) a2.mid; (**c**) a3.mid; (**d**) a4.mid; (**e**) a5.mid; (**f**) a6.mid; (**g**) a7.mid; (**h**) a8.mid; (**i**) a9.mid; (**j**) a10.mid; (**k**) b1.mid; (**l**) b2.mid; (**m**) b3.mid; (**n**) b4.mid; (**o**) b5.mid; (**p**) b6.mid; (**q**) b7.mid; (**r**) b8.mid; (**s**) b9.mid; (**t**) b10.mid; (**u**) c1.mid; (**v**) c2.mid; (**w**) c3.mid; (**x**) c4.mid; (**y**) c5.mid; (**z**) c6.mid; (**aa**) c7.mid; (**ab**) c8.mid; (**ac**) c9.mid; (**ad**) c10.mid.

In our experiments, the second columns of Tables 2, 3 and 4 depict the thirty produced URLs, and the third columns of Tables 2, 3 and 4 illustrate the thirty corresponding strings of pitches. The given relationship between characters and pitches is given in Table 5. Furthermore, the thirty generated MIDI files are shown in the fourth columns of Tables 2, 3 and 4. The thirty music scores of these MIDI files are illustrated in Fig. 7.

**Table 2:** The relationship between a URL and its string of pitches when each URL has ten characters.

No	URL	String of pitches	Midi file produced by Overture
1	www.edu.cn	F7 F7 F7 C4 B4 A4 D7 C4 G4 D6	a1.mid
2	www.sfw.cn	F7 F7 F7 C4 B6 C5 F7 C4 G4 D6	a2.mid
3	www.blg.cn	F7 F7 F7 C4 F4 B5 D5 C4 G4 D6	a3.mid
4	www.wai.cn	F7 F7 F7 C4 F7 E4 F5 C4 G4 D6	a4.mid
5	www.nt.com	F7 F7 F7 C4 D6 C7 C4 G4 E6 C6	a5.mid
6	www.sby.cn	F7 F7 F7 C4 B6 F4 A7 C4 G4 D6	a6.mid
7	www.yun.cn	F7 F7 F7 C4 A7 D7 D6 C4 G4 D6	a7.mid
8	www.nuo.cn	F7 F7 F7 C4 D6 D7 E6 C4 G4 D6	a8.mid
9	www.edg.cn	F7 F7 F7 C4 B4 A4 D5 C4 G4 D6	a9.mid
10	www.qwe.cn	F7 F7 F7 C4 G6 F7 B4 C4 G4 D6	a10.mid

**Table 3:** The relationship between a URL and its string of pitches when each URL has twenty characters.

No	URL	String of pitches	Midi file produced by Overture
1	www.yjsy.ecnu.edu.cn	F7 F7 F7 C4 A7 G5 B6 A7 C4 B4 G4 D6 D7 C4 B4 A4 D7 C4 G4 D6	b1.mid
2	www.xxgk.fafa.edu.cn	F7 F7 F7 C4 G7 G7 D5 A5 C4 C5 E4 C5 E4 C4 B4 A4 D7 C4 G4 D6	b2.mid
3	www.androidweekly.cn	F7 F7 F7 C4 E4 D6 A4 A6 E6 F5 A4 F7 B4 B4 A5 B5 A7 C4 G4 D6	b3.mid
4	www.fengyunzhibo.com	F7 F7 F7 C4 C5 B4 D6 D5 A7 D7 D6 B7 E5 F5 F4 E6 C4 G4 E6 C6	b4.mid
5	www.news.sjtu.edu.cn	F7 F7 F7 C4 D6 B4 F7 B6 C4 B6 G5 C7 D7 C4 B4 A4 D7 C4 G4 D6	b5.mid
6	www.jwc.fudan.edu.cn	F7 F7 F7 C4 G5 F7 G4 C4 C5 D7 A4 E4 D6 C4 B4 A4 D7 C4 G4 D6	b6.mid
7	hospital.hust.edu.cn	E5 E6 B6 F6 F5 C7 E4 B5 C4 E5 D7 B6 C7 C4 B4 A4 D7 C4 G4 D6	b7.mid
8	fgxk.jiangnan.edu.cn	C5 D5 G7 A5 C4 G5 F5 E4 D6 D5 D6 E4 D6 C4 B4 A4 D7 C4 G4 D6	b8.mid
9	zhaosheng.csu.edu.cn	B7 E5 E4 E6 B6 E5 B4 D6 D5 C4 G4 B6 D7 C4 B4 A4 D7 C4 G4 D6	b9.mid
10	faculty.uestc.edu.cn	C5 E4 G4 D7 B5 C7 A7 C4 D7 B4 B6 C7 G4 C4 B4 A4 D7 C4 G4 D6	b10.mid

**Table 4:** The relationship between a URL and its string of pitches when each URL has thirty characters.

No	URL	String of pitches	Midi file produced by Overture
1	www.pku.edu.cn/department.html	F7 F7 F7 C4 F6 A5 D7 C4 B4 A4 D7 C4 G4 D6 D4 A4 B4 F6 E4 A6 C7 C6 B4 D6 C7 C4 E5 C7 C6 B5	c1.mid
2	www.sjtu.edu.cn/yjy/index.html	F7 F7 F7 C4 B6 G5 C7 D7 C4 B4 A4 D7 C4 G4 D6 D4 A7 G5 A7 D4 F5 D6 A4 B4 G7 C4 E5 C7 C6 B5	c2.mid
3	www.fudan.edu.cn/fdjj/list.htm	F7 F7 F7 C4 C5 D7 A4 E4 D6 C4 B4 A4 D7 C4 G4 D6 D4 C5 A4 G5 G5 D4 B5 F5 B6 C7 C4 E5 C7 C6	c3.mid
4	kxyjb.csu.edu.cn/zscqyjszy.htm	A5 G7 A7 G5 F4 C4 G4 B6 D7 C4 B4 A4 D7 C4 G4 D6 D4 B7 B6 G4 G6 A7 G5 B6 B7 A7 C4 E5 C7 C6	c4.mid
5	postdoctor.xmu.edu.cn/main.htm	F6 E6 B6 C7 A4 E6 G4 C7 E6 A6 C4 G7 C6 D7 C4 B4 A4 D7 C4 G4 D6 D4 C6 E4 F5 D6 C4 E5 C7 C6	c5.mid
6	web.zhongxi.cn/xqzl/ldjs/fyzy/	F7 B4 F4 C4 B7 E5 E6 D6 D5 G7 F5 C4 G4 D6 D4 G7 G6 B7 B5 D4 B5 A4 G5 B6 D4 C5 A7 B7 A7 D4	c6.mid
7	www.ustb.edu.cn/hzjl/index.htm	F7 F7 F7 C4 D7 B6 C7 F4 C4 B4 A4 D7 C4 G4 D6 D4 E5 B7 G5 B5 D4 F5 D6 A4 B4 G7 C4 E5 C7 C6	c7.mid
8	www.whu.edu.cn/shfw/zwyjjg.htm	F7 F7 F7 C4 F7 E5 D7 C4 B4 A4 D7 C4 G4 D6 D4 B6 E5 C5 F7 D4 B7 F7 A7 G5 G5 D5 C4 E5 C7 C6	c8.mid
9	www.njust.edu.cn/xxjj/list.htm	F7 F7 F7 C4 D6 G5 D7 B6 C7 C4 B4 A4 D7 C4 G4 D6 D4 G7 G7 G5 G5 D4 B5 F5 B6 C7 C4 E5 C7 C6	c9.mid
10	www.ouc.edu.cn/yldxjs/list.htm	F7 F7 F7 C4 E6 D7 G4 C4 B4 A4 D7 C4 G4 D6 D4 A7 B5 A4 G7 G5 B6 D4 B5 F5 B6 C7 C4 E5 C7 C6	c10.mid

**Table 5:** The relationship between characters and pitches.

Character	Pitch	Character	Pitch	Character	Pitch	Character	Pitch
.	C4	f	C5	m	C6	t	C7
–	D4	g	D5	n	D6	u	D7
a	E4	h	E5	o	E6	v	E7
b	F4	i	F5	p	F6	w	F7
c	G4	j	G5	q	G6	x	G7
d	A4	k	A5	r	A6	y	A7
e	B4	l	B5	s	B6	z	B7

There are two questions worth study. The first concerns the distance between the two machines. The other is about obstacles such as a baffle or something else between the two machines.

To this end, we set up four different scenarios, as shown in Table 6. The difference between the four scenes lies in the distance between the loudspeaker and the pickup and whether there are obstacles between them. The key point is that the decibels measured at the pickup remain unchanged (at least 30 decibels higher than background noise). As shown in Table 6, the results indicate that all thirty AAQRCs are correctly recognized.

**Table 6:** The result of recognition when one machine plays MIDI files with a loudspeaker and another machine picks up the sound and tries to recognize it using Bideyuanli (the average decibels d1 measured at the pickup remain unchanged, the average decibels d2 measured at the loudspeaker change, and the background noise is d3 decibels) Let t1 = m/n if a URL has n characters and m characters are recognized correctly, as well as t2 = d1-d3 = 30.

MIDI file	Value of t1 (scenario 1) (The distance between the two machines is 1 m and there is no obstacle between them)	Value of t1 (scenario 2) (The distance between the two machines is 3 m and there is no obstacle between them)	Value of t1 (scenario 3) (The distance between the two machines is 5 m and there is one wall between them)	Value of t1 (scenario 4) (The distance between the two machines is 10 m and there is two walls between them)
a1.mid	1	1	1	1
a2.mid	1	1	1	1
a3.mid	1	1	1	1
a4.mid	1	1	1	1
a5.mid	1	1	1	1
a6.mid	1	1	1	1
a7.mid	1	1	1	1
a8.mid	1	1	1	1
a9.mid	1	1	1	1
a10.mid	1	1	1	1
b1.mid	1	1	1	1
b2.mid	1	1	1	1
b3.mid	1	1	1	1
b4.mid	1	1	1	1
b5.mid	1	1	1	1
b6.mid	1	1	1	1
b7.mid	1	1	1	1
b8.mid	1	1	1	1
b9.mid	1	1	1	1
b10.mid	1	1	1	1
c1.mid	1	1	1	1
c2.mid	1	1	1	1
c3.mid	1	1	1	1
c4.mid	1	1	1	1
c5.mid	1	1	1	1
c6.mid	1	1	1	1
c7.mid	1	1	1	1
c8.mid	1	1	1	1
c9.mid	1	1	1	1
c10.mid	1	1	1	1

Now, the decibels measured at the loudspeaker remain unchanged, and the decibels measured at the pickup change. Let us see what happens. This time, the results are somewhat different, as depicted in Table 7.

**Table 7:** The result of recognition when one machine plays MIDI files with a loudspeaker and another machine picks up the sound and tries to recognize it using Bideyuanli (the average decibels d1 measured at the loudspeaker remain unchanged, the average decibels d2 measured at the pickup change, and the background noise is d3 decibels) Let t1 = m/n, if a URL has n characters and m characters are recognized correctly, as well as t2 = d2-d3.

MIDI file	Value of t1 \ t2 (scenario 1) (The distance between the two machines is 1 m and there is no obstacle between them)	Value of t1 \ t2 (scenario 2) (The distance between the two machines is 3 m and there is no obstacle between them)	Value of t1 \ t2 (scenario 3) (The distance between the two machines is 5 m and there is one wall between them)	Value of t1 \ t2 (scenario 4) (The distance between the two machines is 10 m and there is two walls between them)
a1.mid	1 \ 43	1 \ 31	1 \ 27	0.60 \ 13
a2.mid	1 \ 46	1 \ 35	1 \ 25	0.60 \ 16
a3.mid	1 \ 42	1 \ 33	1 \ 25	0.70 \ 23
a4.mid	1 \ 45	1 \ 30	1 \ 25	0.60 \ 17
a5.mid	1 \ 42	1 \ 35	1 \ 27	0.50 \ 7
a6.mid	1 \ 37	1 \ 32	1 \ 30	0.70 \ 15
a7.mid	1 \ 37	1 \ 32	1 \ 26	0.50 \ 20
a8.mid	1 \ 39	1 \ 32	1 \ 19	0.40 \ 16
a9.mid	1 \ 37	1 \ 34	1 \ 31	0.50 \ 17
a10.mid	1 \ 43	1 \ 36	1 \ 23	0.40 \ 15
b1.mid	1 \ 49	1 \ 38	1 \ 21	0.45 \ 13
b2.mid	1 \ 47	1 \ 34	1 \ 24	0.65 \ 16
b3.mid	1 \ 40	1 \ 31	1 \ 23	0.40 \ 16
b4.mid	1 \ 41	1 \ 33	1 \ 28	0.40 \ 12
b5.mid	1 \ 45	1 \ 31	1 \ 20	0.45 \ 12
b6.mid	1 \ 42	1 \ 29	1 \ 22	0.45 \ 9
b7.mid	1 \ 46	1 \ 31	1 \ 24	0.50 \ 15
b8.mid	1 \ 47	1 \ 32	1 \ 22	0.45 \ 13
b9.mid	1 \ 48	1 \ 35	1 \ 25	0.65 \ 12
b10.mid	1 \ 33	1 \ 22	1 \ 20	0.85 \ 18
c1.mid	1 \ 44	1 \ 34	1 \ 24	0.70 \ 15
c2.mid	1 \ 42	1 \ 34	1 \ 28	0.43 \ 13
c3.mid	1 \ 44	1 \ 28	1 \ 22	0.56 \ 15
c4.mid	1 \ 48	1 \ 35	1 \ 24	0.50 \ 16
c5.mid	1 \ 45	1 \ 38	1 \ 29	0.60 \ 18
c6.mid	1 \ 43	1 \ 33	1 \ 31	0.53 \ 16
c7.mid	1 \ 43	1 \ 35	1 \ 30	0.50 \ 14
c8.mid	1 \ 43	1 \ 35	1 \ 28	0.53 \ 14
c9.mid	1 \ 43	1 \ 31	1 \ 25	0.40 \ 14
c10.mid	1 \ 41	1 \ 31	1 \ 22	0.53 \ 15

**Figure 8:**
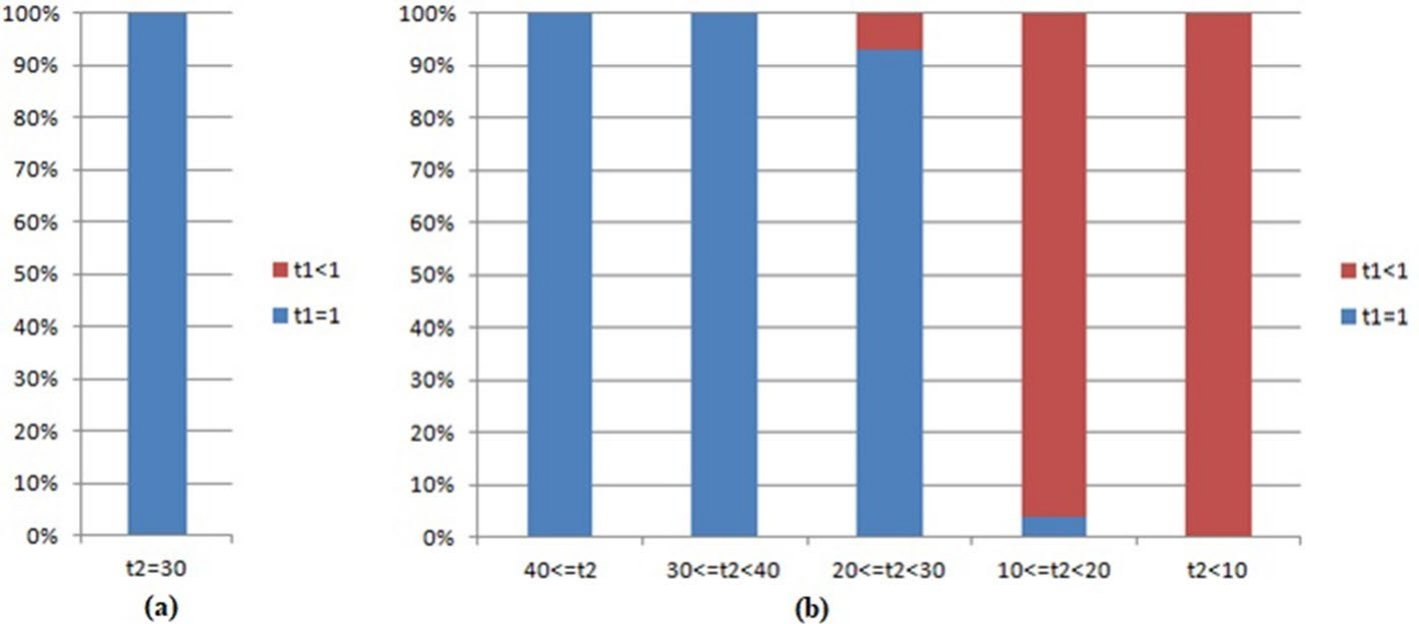
The proportion of the strings of pitches which are correctly identified in the all strings of pitches (a string of pitches is correctly identified, if the corresponding value of t1 is 1). (**a**) in Table 6; (**b**) in Table 7.

Figure 8 summarizes the results of Tables 6 and 7. The relative sound volume is defined as the sound volume at the pickup minus the volume of background noise. If the relative sound volume at the pickup is not less than 30 decibels, all strings of pitches can be correctly and completely identified. This conclusion has nothing to do with the following factors: the length of the string of pitches, the distance between the pickup and the loudspeaker, and whether there are obstacles between the pickup and the loudspeaker. In contrast, if the relative sound volume at the pickup is lower than 30 decibels, the accuracy of recognition of strings of pitches will decrease sharply with decreasing decibels. In other words, the relative sound volume is the only factor affecting the accuracy of recognition. The process of recognition will not be contaminated or affected by environmental noise or obstacles if the difference between the sound volume at the pickup and that of noise is not lower than 30 decibels.

Furthermore, considering that acoustic scene classification (ASC)^19^ is important to reduce noise, we can use it to try and make an AAQRC work in the background of larger noise, without a greater sound volume of AAQRC playback.

## Comparisons between this work and related ones

### Comparison with other acoustic-based approaches

Some great works have been conducted in the field of QR codes related to acoustics.

An approach called acoustic QR codes and differing from the new approach was presented in^1^. Table 8 provides some differences between the two methods.

**Table 8:** Some key differences between the method in Ref. **1** and the new one.

Questions for the candidates	Answers for the new method (the one in Ref. **1**)
Is a generated QR code an image/picture?	No, it is an audio (No)
How to announce or show a generated QR code?	Just play this audio (Um, it is sort of complicated …)
How to scan a generated QR code?	All a user needs to do is open his or her microphone (Um, it is sort of complicated …)
What’s the principle of your QR generation?	Convert a string into pitches, and produce an audio (Um, it is sort of complicated, and it is a long story…)
What’s the principle of your QR recognition?	Read pitches from the audio, and convert them into a string (Um, it is sort of complicated, and it is a long story …)
Can your QR work if the announcer and scanner are far away from each other?	Yes (No)
Can your QR work if there are obstacles between the announcer and scanner?	Yes (Hard to say)

The information in acoustic QR codes is difficult to correctly identify when the distance between the loudspeaker and the pickup reaches 2 m^1^. In contrast, an AAQRC scanner (with a pickup) can correctly identify an URL sent by an AAQRC announcer from 10 m away. According to the above experimental results, we have a reason to believe that the new method can still achieve this even if the distance is larger, as long as the relative sound volume stays at 30 decibels or more.

In addition, Ref. **1** does not report whether the existing method based on acoustic QR codes works if there is an obstacle between the announcer and the scanner. In contrast, an AAQRC scanner (with a pickup) can correctly identify a URL sent by an AAQRC announcer, even if there are two obstacles between the announcer and the scanner. According to the above experimental results, we have a reason to believe that the new method can still achieve this even if more obstacles are present, as long as the relative sound volume stays at 30 decibels or more.

These comparisons highlight the advantages of the new method. The reason is that the new method carries users’ information via sounds that can be heard by humans. In contrast, the approach in Ref. **1** embeds faint inaudible acoustic signals expressing users’ information into an MCLT so the acoustic signals expressing users’ information become background noise, which is covered by the MCLT. This is the fundamental difference between the method in Ref. **1** and the new one. This difference leads to the advantages of the new method.

Audio data transmission (ADT) is a method that sends a message signal through aerial space as a sound^6–9^. Mehrabi et al. found that ADT provides a rapid means of transferring data, in contrast to Bluetooth and image-based QR methods, while requiring minimal physical effort and user coordination^8^. This is the advantage of ADT compared with Bluetooth and image-based QR methods. In fact, ADT is the basis of acoustic-based QR technique. Thus, acoustic-based QR methods have the same advantages compared to image-based QR methods. However, just as inventing an image sensor does not mean inventing an image-based QR technique, although an image-based QR code transmits data through an image sensor, proposing the ADT technique also does not mean proposing the acoustic-based QR technique, although an acoustic-based QR code transmits data via ADT. If ADT was discussed in Ref.^6–9^, this paper and Ref. **1** are talking about an acoustic-based QR technique.

In addition, the experimental scenarios in Ref.^6^ are similar to those in Ref.^1^, and no scenario was tested when the distance between the transmitter and receiver is more than one meter. In contrast, the new method can complete its task even if the distance grows tenfold, prompting the advantage of the new method again.

Chung proposed the effective short-distance transmission of advertisements for smart devices using high frequencies that are not audible to humans^10^. However, these high frequencies only form some trigger signals that enable a smart device to execute a process of advertisement transmission. The advertisement itself is transmitted via a wi-fi network rather than an acoustic channel. Thus, the means in Ref.^10^ is an image-based QR code rather than an acoustics-based QR code, although the traditional former technique is developing in the direction of artistry and robustness^11^.

In short, a number of related works have occurred, and they are important and significant, whereas the proposed approach in this paper is different.

### Comparison with image-based approaches

Currently, the image-based QR method is the popular QR technique, complementing the proposed technique.

First, let us consider security, as shown in Table 9.

**Table 9:** Comparison of security between the image-based technique and the new technique.

Some properties associated with security	The image-based technique	The new one
A legal QR code is replaced covertly by a fake one	Easy	Difficult
Can the above problem be fixed by combining with the block-chain?	Easy	Absolutely
A QR code itself has some virus	Hard	Never
The type/size of the file releasing QR code	Image file/more than dozens of Kbytes	MIDI file/more than hundreds of bytes

A scanning user does not know all the information of every black dot and white dot in a QR image. If the URL is tampered with by a hacker and some information in the black and white dots are altered, the user does not know this. Thus, a legal image-based QR code can be replaced covertly by a fake code. If the proposed method is used, what a user feels is music consisting of a string of pitches, not an image consisting of a large number of black dots and white dots. For the user, it is easy to realize that the music has been changed if a hacker replaces the real URL with a fake URL covertly. Which is easier to perceive, a piece of music is off-key, or a few dots are modified in a large number of black and white dots gathering together irregularly? The answer is obvious. That is why the new method is more effective in terms of combating tampering attacks.

Considering that a single block can store only one Mbyte at most and that some aesthetic QR images have several Mbytes, one can hardly expect the block-chain to help these aesthetic image-based QR codes combat tampering attacks. In contrast, an AAQRC MIDI file has only 1 Kbyte when a URL has one hundred characters. Thus, the block-chain will be useful in terms of dealing with tampering attacks if the proposed method rather than image-based QR methods is employed.

It is generally known that a QR image itself has little ability for a virus due to the number of black and white dots. However, it is difficult for a user to establish a one-to-one map between each of these dots and each of the characters in a URL, and they are not equal in number. That is, some dots do not carry any URL information. Thus, the following possibility cannot be ruled out: a hacker employs some “redundant” dots to carry malware code covertly. In contrast, it is absolutely impossible for a piece of AAQRC music to carry a virus because each character in a URL is mapped to a pitch in a string. That is, a user will find that the music becomes longer so that he or she will be aware of something abnormal if any virus information is embedded.

Second, robustness is also important.

In short, the recognition effect of image-based QR code will be poor if the light is too weak, while the recognition effect of acoustic-based AAQRC code will be poor if there is too much noise. For example, a QR image cannot be recognized in an air-gapped way at an outdoor location without enough light at night, while AAQRC music is hard to recognize in an air-gapped way on a busy street.

Let us consider some extremely significant real-world scenarios as potential applications. Sometimes, you have to join a queue to scan a QR code and keep others at a distance before entering an indoor place. Such real-world scenes are very common in China's COVID-19 epidemic prevention and control, especially in a very large number of railway stations, hospitals, sites of very large-scale nucleic acid testing, and other public places all over the country. In this situation, how to assist people with security via QR conveniently, if you cannot expect a person to scan an image-based QR at night, in the rain, or under the blazing sun?

Of course, an image-based QR can also be used if a few black and white dots are blurred, whereas an AAQRC cannot be used if one pitch is inaccurate. The reason is that a QR image contains some redundant information, whereas no redundancy occurs in an AAQRC. Thus, this is an advantage rather than disadvantage of AAQRC. Furthermore, this problem does not need to be considered in many practical cases. For example, a source with unified authentication will easily eliminate any inaccurate pitch in the real-world scenarios mentioned above, which are relevant to COVID-19 epidemic prevention and control, in a potential application.

Third, let us think about artistry.

Which will make users comfortable? An image-based QR, or the acoustic-based AAQRC? Ordinary QR codes present two colors: black and white. To improve the artistry of a QR, our lab put forward a sort of aesthetic-based QR technique^11^, called “Meiyao”^12^, which has played an important role in the control of COVID-19 outbreaks in many cities in Henan Province, China^13^. In fact, Meiyao provides users not only a QR function but also a delightful user experience^11^, due to rich colors and beautiful images, without affecting the robustness. For the method proposed in this paper, we aim to enhance the user experience from the perspective of sound rather than vision. Which one is better? One man's meat is another man's poison!

**Figure 9:**
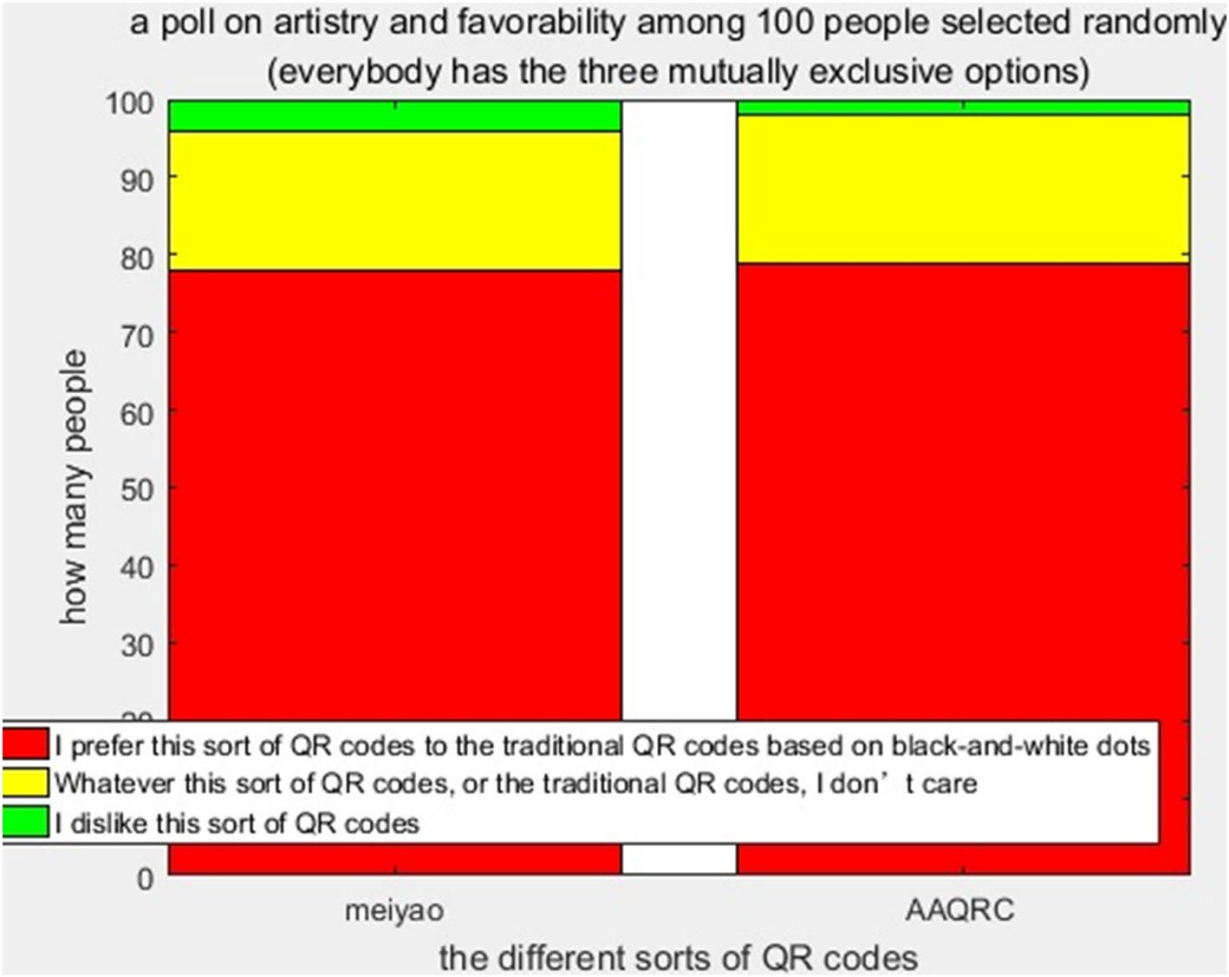
A poll on artistry and favorability among 100 persons selected randomly.

We performed a test. A poll on artistry and favorability among 100 students selected randomly at Zhoukou Normal University was made. To ensure fairness, the selected students were majoring in science and engineering, which had nothing to do with music, painting and art. Everyone evaluated Meiyao and AAQRC independently and, respectively, according to his or her own feelings, after using a given group of the prototype of Meiyao codes and prototype of AAQRC codes. Everybody has the following three mutually exclusive options: “I prefer this sort of QR code (Meiyao or AAQRC) to traditional QR codes based on black and white dots”, “whatever this sort of QR code (Meiyao or AAQRC), or traditional QR codes based on black and white dots, I don’t care”, and “I dislike this sort of QR code (Meiyao or AAQRC)”. Figure 9 illustrates the result of this poll. A few more persons prefer AAQRC over Meiyao as his or her favorite, although it is just a tiny gap, indicating that different strokes for different folks.

Fourth, accessibility is vital for users.

There are two ways to access a QR image or AAQRC music: air-gap access and local access. On the AAQRC side, they are Mode 2 and Mode 1, respectively. In the former mode, a transmitter displays images or plays sounds, and the visual signals of the images and the acoustic signal of the sounds travel through the air before they are received by a receiver. In the latter mode, neither visual signals in terms of images nor acoustic signals in terms of sounds travel through the air, so the receiver only needs to recognize a QR image or AAQRC music on the local machine. Thus, we only need to consider the former way when we talk about accessibility. Table 10 provides some comparisons.

**Table 10:** Comparison of accessibility and robustness between the image-based technique and the new technique.

Some properties associated with accessibility	The image-based technique	The new one
Can image/sound be displayed/played via internet/TV?	Yes	Yes
Can image/sound be displayed/played via radio?	No	Yes
Can image/sound be displayed/played via very large screens at a large-scale site?	Yes	No
Can image/sound be displayed/played via high-power loudspeakers at a large-scale site?	No	Yes
What about the speed when image/sound is displayed/played?	Fast	Slow, in theory Faster, on the occasion of queuing
Can image/sound be displayed/played at night, in the rain, or blazing sunshine?	No	Yes

For example, on a campus or in a shopping mall, an AAQRC will be more suitable than an existing image-based QR if a QR code needs to be put on the market in a large-scale and nondirectional way. The reason for this is that high-power loudspeakers are more common than very large screens at the real-world scenes of a campus or the indoor space of a shopping mall.

In terms of accessibility, speed needs special attention. In theory, AAQRC is slower than the image-based QR methods because listening to a piece of music expressing an AAQRC takes more time than scanning a traditional QR image. However, the reality may be somewhat different in many cases. We performed another test, as follows.

The 100 persons mentioned above lined up outside, waiting to enter an indoor space. Everyone needs to “scan” a QR code before entering the door. There are two optional “scanning” ways: one is to scan an image-based QR code, and the other is to use an AAQRC. Our test results show that 14 persons enter the door in one minute on average, using the former way. In contrast, 16 persons enter the door in one minute on average using the latter way. Clearly, an AAQRC is not slower than traditional image-based QR in this test. The reason is that even if you are further in the queue, you can hear the music expressing the AAQRC and can complete the process of AAQRC "scanning". In contrast, you must go to the front of the queue, i.e., wait for the queue to move until you arrive at the entrance of the room to complete the process of traditional QR scanning.

We take COVID-19 epidemic prevention and control as an example of a potential application. Supposing that a real-world scene with a queue is relevant to COVID-19 epidemic prevention and control, the fact mentioned in the previous paragraph can help us realize that speed is not an obstacle for an AAQRC in some vital real-world scenarios, compared to image-based QR methods. Of course, multi-play can disturb AAQRC recognition. However, any multi-play will be prohibited in such an extremely significant real-world scenario. As a result, this problem can be solved easily.

As analyzed above, the new method has some advantages and limitations compared with the image-based QR technique. In terms of shortcomings and limitations, AAQRC music is difficult to recognize in an air-gapped way in a busy street, as mentioned above. In addition, it will take a relatively long time to play an AAQRC once in some scenarios if the corresponding URL has too many characters.

In summary, what matters is a combination of security, robustness, artistry and accessibility. We can safely say that the image-based approaches and the newly proposed approach complement each other, according to the comprehensive analysis, tests and comparisons mentioned above. It should be noted that we do not think the new method is superior to the existing ones in terms of all the metrics. So what? It is not necessary to let the new method achieve this goal.

Some studies are relevant to sound, images and QR functions. For example, Sarkar et al. presented an interesting approach for tackling multiple QR codes all at once, and some multimedia data, including text, images, and audio data, can be converted to QR codes^17^. However, the generated QR objections waiting for scanning still exist in some PDF files or printed papers. Thus, this method is an image-based QR method, rather than an acoustical-based QR method.

### More related works

Next, we will briefly survey a bigger picture or roadmap.

There were some early works^20,21^ using audible acoustic signals for wireless communications. However, their ranges did not exceed 0.5 m, causing these methods to be considered near-field communication rather than QR codes. Furthermore, another method implements communication by embedding messages in audible audio^22^. However, the high frequency sound used is particularly sharp, and it lies beyond the scope of the frequencies of sound that people often hear in daily life. As a result, this method is a great one for short-range communications on some occasions, but it is not suitable for QR codes for daily use.

For an image-based QR, there have been many studies in recent years, including but not limited to the following.

First, readability (robustness) is very important to a QR image. Deformation may reduce the readability of a QR image. To this end, Ref.^23^ proposed a method to embed QR codes onto freeform surfaces using a low-end consumer-level 3D printer when deformation of QR images is caused by object surfaces that are not flat. Refs.^24,27^ also introduced some methods to address issues related to deformation and readability. In addition, Ref.^31^ proposed an algorithm for QR images, trying to address out-of-focus problems, which has an impact on QR readability.

Second, QR codes are closely related to some issues of information security, such as secret sharing via QR codes^25,35^, QR security in mobile payments^34^ and QR detection against a malicious URL^26^.

As everyone knows, QR codes are often used to collect data, which may lead to the issue of data privacy in some cases. More broadly, how do we realize a good tradeoff between the availability of data and privacy preservation for data in several fields in course of data processing? Prof. Qi proposed some illuminating approaches^43–45^, providing great insights into the above question.

Third, some extended forms of QR codes have occurred, aiming to meet various real-world requirements, such as dual-modulated QR codes for proximal privacy and security^28^ and “Meiyao” for QR artistic quality^11,12,30^. It should be noted that something interesting has happened. For example, black modules in standard QR codes can be replaced by specific texture patterns^32^, and a URL can be obtained by decoding a common picture that seems to have nothing to do with QR^33^. Furthermore, 3D^37^ and 4D QR codes^36^ have already been developed, although traditional QR codes are considered to be essentially 2D matrix images.

Fourth, QR images need to be presented on a microscopic scale^29^ in some situations. A State of the art technique can inscribe a QR code composed of a set of 25 × 25 microdots, and each microdot has a diameter of approximately 14 µm^38^. In fact, a QR code can be integrated into a microdevice with a size of hundreds of microns^39^. In addition, a material method for micro QR codes has also been discussed^40^.

Fifth, the application of QR codes is always a research focus. To date, this technique has been applied to not only life but also various fields of science, such as optical retrieval^41^ and taxonomy of species^42^.

## Conclusions

Audible sound made by humans, except for natural language, such as an infant cry, can convey a certain message^18^. The newly proposed method carries and transfers URL information with a kind of artificial audible sound outside natural language, i.e., piano music. On the one hand, no QR image is generated. On the other hand, it is possible to “scan” such a QR sound remotely even if there are obstacles between the QR announcer (loudspeaker) and QR scanner (pickup). Both are benefits of using the new approach. Clearly, these characteristics establish that the new method is more practical than existing acoustic QR methods and complements existing image-based QR methods, implying the prospects for future applications of the new approach in practice.

## Data Availability

All data generated or analyzed during this study are included in this published article.

## References

[1] Dagan I, Binyamin G, Eilam A (2017). Delivery of QR codes to cellular phones through data embedding in audio. Int. Conf. Sci. Electr. Eng..

[2] https://www.overturechina.com/

[3] https://www.midieditor.org/

[4] https://bideyuanli.com/pp

[5] https://baike.baidu.com/item/Sound%20Meter/7583837?fr=aladdin

[6] Cueva, Y., Castro, H., Barrientos, A. *et al.* Comparative analysis of technologies for audio data transmission. IEEE Sci. Human. Int. Res. Conf. IEEE Press (2018)

[7] Wu S, Huang J, Huang D (2005). Efficiently self-synchronized audio watermarking for assured audio data transmission. IEEE Trans. Broadcast..

[8] Mehrabi A, Mazzoni A, Jones D (2019). Evaluating the user experience of acoustic data transmission. Pers. Ubiquit. Comput..

[9] Isnawati, A. F., Citra, V. O., Hendry, J. Performance Analysis of Audio Data Transmission on FBMC - Offset QAM System. In *2019 IEEE International Conference on Industry 4.0, Artificial Intelligence, and Communications Technology (IAICT)*, BALI, Indonesia, 2019, pp. 81–86, 10.1109/ICIAICT.2019.8784810.

[10] Chung MB (2016). Effective near advertisement transmission method for smart-devices using inaudible high-frequencies. Multimed. Tools Appl.

[11] Xu M (2019). Stylized Aesthetic QR Code. IEEE Trans. Multimed..

[12] An ultimate one-stop management platform for generating and beautifying QR codes, https://www.meiyaoma.com/, 2018 (in Chinese)

[13] "Henan’s QR codes for health" which was independently developed by the research team at Zhengzhou University, has been applied in the whole province, http://www5.zzu.edu.cn/yqfk/info/1003/1608.htm, March, 2020 (in Chinese).

[14] How to read a MIDI file, https://www.jianshu.com/p/31d02765e1ec, Feb, 2019. (in Chinese)

[15] Illustration on principle: harmonics and their formation, https://bideyuanli.com/p/3238, April, 2014. (in Chinese)

[16] Pitches, https://bideyuanli.com/p/3673, September, 2014. (in Chinese)

[17] Sarkar, S., Pu, L., Wu, H., Huang, S. C., Wu, Y. New multimedia archiving technique using multiple quick-response codes. 2017 IEEE International Symposium on Broadband Multimedia Systems and Broadcasting (BMSB), Cagliari, pp. 1-6, 10.1109/BMSB.2017.7986236 (2017)

[18] Liu L, Li W, Wu X, Zhou BX (2019). Infant cry language analysis and recognition: an experimental approach. IEEE/CAA J. Automatica Sinica.

[19] Ren Z, Qian K, Zhang Z, Pandit V, Baird A, Schuller B (2018). Deep scalogram representations for acoustic scene classification. IEEE/CAA J. Automatica Sinica.

[20] Nandakumar R, Chintalapudi KK, Padmanabhan VN (2013). Dhwani: Secure peer-to-peer acoustic NFC. Comput. Commun. Rev..

[21] Zhang B (2014). PriWhisper: Enabling keyless secure acoustic communication for smartphones. IEEE Internet of Things J..

[22] Wang, Q., Ren, K., Zhou, M., *et al.* Messages behind the sound: Real-time hidden acoustic signal capture with smartphones, ACM MobiCom, New York, NY, USA, pp. 29–41 (2016).

[23] Papp G, Hoffmann M, Papp I (2021). Improved embedding of QR codes onto surfaces to be 3D printed. Comput. Aided Des..

[24] Papp G, Hoffmann M, Papp I (2022). Embedding QR code onto triangulated meshes using horizon based ambient occlusion. Comput. Graph. Forum..

[25] Huang PC, Chang CC, Li YH (2021). Enhanced (n, n)-threshold QR code secret sharing scheme based on error correction mechanism. J. Inf. Sec. Appl..

[26] Wahsheh Heider, A. M.; Al-Zahrani Mohammed, S. Secure real-time computational intelligence system against malicious QR code links. *Int. J. Comput. Commun. Control*, 2021. 10.15837/ijccc.2021.2.4xyz

[27] Eugênio Gonçalves H, Xavier Medeiros L, Coutinho Mateus A (2021). Algorithm for locating the vertices of a QR code and removing perspective. IEEE Latin Am. Trans..

[28] Barron I, Yeh HJ, Dinesh K (2020). Dual modulated QR codes for proximal privacy and security. IEEE Trans. Image Process..

[29] Liu H, Chou T, Lu C (2021). Improving readability by modifying graphic QR code microstructure. Electron. Lett..

[30] Xu M, Li Q, Niu J (2021). ART-UP: A novel method for generating scanning-robust aesthetic QR codes. ACM Trans. Multimedia Comput. Commun. Appl. (TOMM).

[31] Chen R, Zheng Z, Pan J (2021). Fast blind deblurring of QR code images based on adaptive scale control. Mobile Netw. Appl..

[32] Yu L, Cao G, Tian H (2021). Recognition of printed small texture modules based on dictionary learning. J. Image Video Proc..

[33] Zhang P (2021). VisCode: Embedding information in visualization images using encoder-decoder network. IEEE Trans. Visual. Comput. Graph..

[34] Zhou Y, Hu B, Zhang Y (2021). Implementation of cryptographic algorithm in dynamic QR code payment system and its performance. IEEE Access.

[35] Xiong L, Zhong X, Xiong NN (2020). QR-3S: A high payload QR code secret sharing system for industrial Internet of Things in 6G networks. IEEE Trans. Industr. Inf..

[36] Chen L, Zhang Y, Ye H (2021). Color-changeable four-dimensional printing enabled with ultraviolet-curable and thermochromic shape memory polymers. ACS Appl. Mater. Interfaces..

[37] Peng H, Lu L, Liu L (2019). Fabricating QR codes on 3D objects using self-shadows. Computer-Aided Des..

[38] Batista AJ, Vianna PG, Ribeiro HB (2019). QR code micro-certified gemstones: femtosecond writing and Raman characterization in Diamond, Ruby and Sapphire. Sci. Rep..

[39] Zhang C, Hu Y, Du W (2016). Optimized holographic femtosecond laser patterning method towards rapid integration of high-quality functional devices in microchannels. Sci. Rep..

[40] Polito G, Robbiano V, Cozzi C (2017). Template-assisted preparation of micrometric suspended membrane lattices of photoluminescent and non-photoluminescent polymers by capillarity-driven solvent evaporation: Application to microtagging. Sci. Rep..

[41] Wang X, Chen W, Mei S (2015). Optically secured information retrieval using two authenticated phase-only masks. Sci. Rep..

[42] Gogoi B, Wann SB, Saikia SP (2020). DNA barcodes for delineating Clerodendrum species of North East India. Sci. Rep..

[43] Kong L, Wang L, Gong W, Yan C, Duan Y, Qi L (2021). LSH-aware multitype health data prediction with privacy preservation in edge environment. World Wide Web.

[44] Qi L, Hu C, Zhang X (2020). Privacy-aware data fusion and prediction with spatial-temporal context for smart city industrial environment. IEEE Trans. Industr. Inf..

[45] Qi L, Wang X, Xu X (2020). Privacy-aware cross-platform service recommendation based on enhanced locality-sensitive hashing. IEEE Trans. Netw. Sci. Eng..

